# Cancer Patients’ Experiences with Telehealth before and during the COVID-19 Pandemic in British Columbia

**DOI:** 10.3390/curroncol29060335

**Published:** 2022-06-10

**Authors:** Sara Izadi-Najafabadi, Lisa McQuarrie, Stuart Peacock, Ross Halperin, Leah Lambert, Craig Mitton, Helen McTaggart-Cowan

**Affiliations:** 1 Department of Cancer Control Research, BC Cancer Research Institute, Vancouver, BC V5Z 1L3, Canada; lmcquarrie@bccrc.ca (L.M.); speacock@bccrc.ca (S.P.); hcowan@bccrc.ca (H.M.-C.); 2 Canadian Centre for Applied Research in Cancer Control, Vancouver, BC V5Z 1L3, Canada; 3 Faculty of Health Sciences, Simon Fraser University, Burnaby, BC V5A 1S6, Canada; 4 BC Cancer—Kelowna, Kelowna, BC V1Y 5L3, Canada; rhalperin@bccancer.bc.ca; 5 Department of Nursing and Allied Health Research and Knowledge Translation, BC Cancer, Vancouver, BC V5Z 1G1, Canada; leah.lambert@bccancer.bc.ca; 6 School of Nursing, University of British Columbia, Vancouver, BC V6T 2B5, Canada; 7 Centre for Clinical Epidemiology and Evaluation, Vancouver Coastal Health Research Institute, Vancouver, BC V5Z 1M9, Canada; craig.mitton@ubc.ca; 8 School of Population and Public Health, University of British Columbia, Vancouver, BC V6T 1Z3, Canada

**Keywords:** cancer, COVID-19 pandemic, patient-reported experiences, telehealth

## Abstract

Background: Patients have had their cancer care either postponed or changed to telehealth visits to reduce exposure to COVID-19. However, it is unclear how these changes may have affected their experiences. We aim to identify patient characteristics that affect telehealth experiences and evaluate their preferences for using telehealth in the future. Methods: Patients who completed the Outpatient Cancer Care (OCC) Patient Experience Survey were invited to participate. They comepleted the modified OCC Survey, which focused on telehealth during the pandemic. Linear and logistic regression analyses were used to identify patient characteristics that influenced telehealth experiences and preferences for future telehealth use. Results: Perceived ease of participation in telehealth is a significant predictor of the change in patients’ ratings of their telehealth experience. We found that cancer patients had lower preferences for using telehealth in the future if they were older, female, or non-white; resided in an urban area; had no previous telehealth experience; had lower education; and had poorer mental health. Conclusions: To optimize cancer care and improve equitable access to high-quality telehealth care during the pandemic and beyond, clinicians and policymakers will need to consider patients’ self-reported experiences and their personal characteristics.

## 1. Introduction

During the COVID-19 pandemic, health care systems have been overwhelmed by the number of COVID-19 cases and by shortages of medical supplies and personnel [1,2,3]. The pandemic posed a major risk of compromising treatment for cancer patients around the world [4]. Cancer patients may have modified treatments, canceled screening and surveillance tests, delayed surgical procedures, and increased uncertainty in mapping out future care [5,6].

To ensure care continuity and quality while reducing the risk of COVID-19 exposure, the structure and delivery of care were rapidly redesigned at the start of the pandemic in many jurisdictions in Canada and beyond. Many in-person interactions were transitioned to telehealth, which is the provision of health care remotely by means of telecommunications technology (e.g., telephone, video) [7,8]. Increases in telehealth visits were observed across Canada at the start of the pandemic [9,10,11].

Globally, telehealth appointments have been well received by patients, with high rates of satisfaction among patients [9,10,12,13,14,15,16,17,18,19,20,21]. Benefits of telehealth identified by patients included reduced exposure to COVID-19 [15,22] and convenience (e.g., time and money saved by avoiding travel to in-person appointments) [10,16,17,20,22,23,24]. However, this rapid shift into telehealth has substantial negative consequences in terms of clinician-patient relationships, cybersecurity and technical aspects, and health care accessibility [25]. Cancer patients have expressed difficulty with cultivating a clinician-patient relationship, and clinicians have had concerns regarding the ability to conduct physical examinations and read physical cues in the patient’s body language [12,16,20,23,24,26,27]. Moreover, cancer patients preferred in-person appointments for receiving difficult news [18,20,24]. A small proportion of patients also reported technical issues during telehealth appointments [12,17,22,23].

In this study, we were presented with a unique opportunity to conduct a natural experiment to compare cancer patients’ experiences with telehealth before and during the pandemic in BC. We build on previous work conducted by the BC Ministry of Health (MoH) [28], whose responsibility is to ensure quality, appropriate, cost-effective, and timely health services for individuals in the province. Specifically, the aims of this study were to: (1) identify patient characteristics that affect changes in their telehealth experiences (before and during the pandemic) and (2) evaluate patients’ preferences for using telehealth in the future. 

## 2. Materials and Methods

### 2.1. Study Design and Survey

The MoH developed a 126-item Outpatient Cancer Care (OCC) Patient Experience Survey [29] to inquire about cancer patients’ experiences with treatment, care providers, and telehealth in the previous six months. Additional items asked patients about their diagnosis, treatment, and health-related quality of life (HRQL) (i.e., the Veterans RAND 12 Item Health Survey (VR-12)) [30]. The OCC Survey was administered in two waves to BC patients receiving active cancer treatment at a BC Cancer Centre and/or at a Community Oncology Network (CON) site. Wave 1 was administered October–December 2019 and collected data from patients who received cancer treatment between April and June 2019. Wave 2 was administered January–May 2020 and collected data from patients who received cancer treatment between July and October 2019. Responses to Wave 1 and 2 surveys captured patients’ experiences before the pandemic, but for the purposes of this study, we focused on Wave 2 for pre-pandemic data.

For the current study, patients who completed the OCC Survey Wave 2 were invited to participate in this study between 1 May and 30 July 2021. They completed a modified OCC Patient Experience Survey, hereafter referred to as Wave 3. This modified 62-item survey included sociodemographic and treatment questions, as well as the VR-12. We included additional items designed by the BC Office of Patient-Centred Measurement (BCPCM) [31] to focus on patient experiences with telehealth and their cancer care providers during the COVID-19 pandemic. The responses to Wave 3 described patients’ experiences during the pandemic. Specifically, patient’s experience with telehealth was measured on an 11-point response scale, ranging from 0 (very poor experience) to 10 (very good experience), and the patient’s strength of preference for using telehealth after the pandemic was categorized on a 4-level response scale (i.e., definitely yes, probably yes, probably no, and definitely no). Cancer patients’ experiences and responses to Wave 2 (before the pandemic) and Wave 3 (during the pandemic) are the focus of this study. A copy of the survey is available upon request.

### 2.2. Study Participants

To be eligible for this study, patients needed to self-report having received cancer care during the pandemic. For the purpose of this study, the start of the pandemic is defined as 16 March 2020, the date on which BC Cancer shifted in-person cancer care to virtual care as a result of the pandemic. Patients had the option of completing the Wave 3 survey either online or on paper. The survey was available in English and, upon request, in Punjabi and in traditional and simplified Chinese.

### 2.3. Analysis

#### 2.3.1. Study Sample Description

Using the responses to Wave 3, the study sample was characterized in terms of current age, sex, education, race, health authority, region of residence, previous experience with telehealth, and tumor type. If information was missing, the values reported in Wave 2 were used. Categorical variables were summarized as the proportion of the sample within each group, and continuous variables that were normally distributed were summarized as means and standard deviations (SDs).

Responses to the VR-12 were used to generate each patient’s physical component score (PCS) and mental component score (MCS), where higher scores represent better physical and mental health, respectively [30]. A paired t-test was used to compare the PCS and MCS scores between Waves 2 and 3. The types of resources patients used to seek medical advice before and during the pandemic were collated and compared using a McNemar’s test. The Bonferroni method was used to correct for multiple testing.

#### 2.3.2. Multiple Imputation for Missing Data

Missing information was present for all variables of interest. Missingness ranged from 0.4 to 18%, with the question “Was the care advice you received during your Virtual Health/Telehealth visit(s) helpful to you?” accounting for the most missing values. The results from Little’s missing completely at random (MCAR) test [32] showed that the data were missing completely at random (*X*^2^ = 31727.6, *df* = 33032, *p* = 1.0). As such, multiple imputation models were established for each variable with missing values; binary variables were imputed using logistic regression, and continuous variables were imputed with predictive mean matching. The number of imputed data sets was 30. The imputations were conducted separately for the two outcome measures (i.e., the change in the ratings of patients’ experience with telehealth and patients’ preferences for the future use of telehealth) because the outcomes applied to different subgroups.

#### 2.3.3. Experiences with Telehealth

The ordinal variables of telehealth ratings for Waves 2 and 3 were summarized as medians, and a Wilcoxon signed-rank test was used to assess whether patients’ experiences differed between the two time points. The following independent variables were used in a multivariate linear regression as they were anticipated to influence a change in patients’ telehealth ratings: current age, sex, race, region of residence, health authority where care was received, education level, perceived ease of participating in a telehealth consult, and VR-12 score (i.e., PCS and MCS).

#### 2.3.4. Preference to Use Telehealth in the Future

Patient responses to preferences for using telehealth in the future were collapsed to “yes” (i.e., definitely yes and probably yes) and “no” (i.e., definitely no and probably no) to improve interpretation and to manage any quasi-complete separation in the logistic regression. The same independent variables used in the multivariate linear regression plus previous experience with telehealth were included in this logistic regression to determine factors affecting patients’ preferences to use telehealth when the COVID-19 pandemic is over.

Statistical tests of associations were completed using SAS Software, version 9.4 (SAS Institute Inc., Cary, NC, USA). Multiple imputation and regression analyses were conducted in R, version 4.1.2, using the MICE package, version 3.14.0 [33]. Statistical significance was defined at *p* ≤ 0.05.

## 3. Results

### 3.1. Study Participants

Of the cancer patients who completed Wave 2 (*n* = 5347), *n* = 614 were not sampled because they were deceased as of 30 April 2021 (*n* = 284) or they were deemed ineligible to participate (e.g., who received care from a health authority that did not grant permission to re-contact their patients or who were identified to be receiving end-of-life care) (*n* = 330). Therefore, *n* = 4733 participants were invited to participate in this study.

A total of 2623 cancer patients completed the Wave 3 survey, for a response rate of 0.55. Of those patients completing Wave 3, the following were excluded because they self-reported not receiving care for their cancer during the pandemic (*n* = 631) or did not provide any information regarding their receipt of cancer care during the pandemic (*n* = 21): Two patients completed the survey twice; the duplicate responses were removed randomly. The final analysis set included 1958 cancer patients (Table 1). The mean (±SD) age of the patients was 69.4 (±10.9) years. The majority of the sample were female (assigned at birth; *n* = 1074, 55%) and white (*n* = 1533, 78%).

The patients’ mean (±SD) MCS significantly increased during the pandemic (53.02 (±10.79)) compared with before the pandemic (50.78 (±9.99)) (*t* = 10.13, *df* = 1886, *p* < 0.0001). The mean (±SD) PCS of the study patients significantly decreased during the pandemic (41.20 (±11.85)) compared with before the pandemic (42.86 (±10.83)) (*t* = −7.9, *df* = 1895, *p* < 0.0001).

### 3.2. Medical Resources Utilized by Patients 

More patients reported participating in at least one virtual health visit during the pandemic (*n* = 1382, 71%) than before the pandemic (*n* = 789, 40%). The frequency of patients who attended at least one phone visit (*X*^2^ = 282.83, *df* = 1, *p* < 0.0001) or video visit (*X*^2^ = 14.94, *df* = 1, *p* < 0.0001) changed significantly during the pandemic compared with before the pandemic (Table 2). 

Regardless of the survey wave, patients reported more often having phone visits with their regular oncologist whom they consulted with most often and their family doctor (Supplementary Figure S1a). However, phone visits with regular oncologists decreased between the survey waves (before pandemic: *n* = 548, 40%; during pandemic: *n* = 947, 36%), whereas the proportion of phone visits with family doctors increased (before the pandemic: *n* = 353, 25%; during the pandemic: *n* = 760, 29%). The proportion of patients with at least one phone visit with their family doctor was significantly different (*X*^2^ = 32.6, *df* = 1, *p* = 0.0001), even after Bonferroni correction (*p* < 0.0025), during the pandemic compared with before the pandemic. 

Patients reported having video visits with their regular oncologists (*n* = 90, 41%) or another oncologist (*n* = 36, 17%) before the pandemic (Supplementary Figure S1b). Although the proportion of video visits with their regular oncologists decreased during the pandemic (*n* = 103, 33%), the proportion of video visits with their family doctors increased (*n* = 65, 21%). However, the proportion of video visits with other types of health care professionals did not change from before to during the pandemic.

### 3.3. Patient Experiences with Telehealth

Of patients who had at least one telehealth visit during the pandemic, most reported positive experiences with telehealth. In particular, the majority found telehealth visits easy or very easy to use (*n* = 927, 80%) and helpful or very helpful (*n* = 891, 79%), and most would probably or definitely recommend telehealth to other patients (*n* = 1032, 87%) (Table 3 and Table 4).

Despite the favorable experiences reported by the patients, a relatively large proportion of patients, with and without telehealth visit experiences during the pandemic, reported that telehealth was not a good alternative to in-person visits (*n* = 657, 36%) and that they would not use telehealth if offered after the COVID-19 pandemic (*n* = 678, 37%).

### 3.4. Patient Characteristics Affecting Telehealth Experiences

Not surprisingly, an increase in the number of patients using telehealth was observed during the pandemic (*n* = 1382) compared with before the pandemic (*n* = 463). Patients’ ratings of their telehealth experiences changed (*W* = −1780, *p* < 0.0001) during the pandemic (median = 9) compared with before the pandemic (median = 10).

Using responses from patients reporting telehealth experience ratings at both time points (*n* = 331), patients’ perceived ease of participation and patients’ education level were identified as significant predictors (Table 5). Patients who perceived participating in telehealth during the pandemic as being at least “neither easy nor difficult” had greater changes in their ratings of telehealth experience from before to during the pandemic compared to patients who perceived participating in telehealth as “difficult or very difficult”. Moreover, patients with higher education levels had smaller changes in their ratings of telehealth experience.

### 3.5. Patient Characteristics Affecting Preferences to Use Telehealth in the Future

Of patients who reported having used telehealth during the pandemic (*n* = 1382), age, sex, previous experience with telehealth, region of residence, education, ease of participation in telehealth visits, and MCS were significant predictors of their preference to use telehealth in the future (Table 6). Specifically, older female patients with an education level of high school or less who live in urban areas, have lower MCS, and perceive telehealth as difficult or very difficult to use had lower preferences for using telehealth if it is offered after the COVID-19 pandemic is over. The results show that patients with previous experience with telehealth are 1.5 times more likely to indicate that they would use telehealth in the future compared with those without this experience. Additionally, patients who perceived participation in telehealth visits as “neither easy nor difficult” and as “easy” are approximately 11.4 and 29.7 times, respectively, more likely to prefer to use telehealth in the future compared with those who reported participating in telehealth to be difficult.

## 4. Discussion

The study findings demonstrated that the proportion of telehealth visits increased during the COVID-19 pandemic; this is consistent with other reports [34]. We found a higher proportion of telehealth visits with oncologists and family doctors compared with other health care professionals (e.g., nurse, psychiatrist, psychologist); this may be a result of a few factors. First, the higher proportion of virtual oncology visits may be a result of a BC pilot project launched in 2018 to deliver follow-up cancer care virtually by oncologists [35]. Second, the College of Family Physicians of Canada developed a framework to improve patients’ cancer care experiences during the pandemic. Specifically, family doctors were encouraged to transition to telehealth, provide supportive care, and collaborate with the cancer care team to ensure the continuity of care [36]. The recommended responsibilities of family doctors may explain the increased frequency in phone visits during the pandemic. Third, telehealth visits with family doctors were covered under public health insurance even before the COVID-19 pandemic, making it easier for patients to access this service [35]. Immediately after the start of the pandemic, public health insurance approved the use of phone visits, which enabled physicians to provide health care via the phone to reduce the risk of COVID-19 exposure while increasing health care accessibility to patients who do not have access to video communication [37].

Similar to the results in Canada and elsewhere [18], we found that phone visits were more commonly used than video visits. This may be a result of different factors, such as organizations’ telehealth infrastructures, patient and clinician preferences, and patients’ demographics (i.e., age, race) including access to technology (e.g., phone vs computer) [27,38]. Phone visits do not require high digital literacy or internet accessibility, whereas video visits may require more IT support and higher network bandwidth, thus making the phone a more equitable and accessible option [39]. The type of telehealth visit (i.e., phone vs video) is not associated with social vulnerability (i.e., socioeconomic status, transportation, disability, unemployment) [27], or patients’ satisfaction with their care [40,41]. Previous studies have stated that both patients and health care providers prefer video over the phone as it allows for better nonverbal communication and clinician-patient relationships [10,16,17,18,42] To support these preferences, health organizations can improve their patients’ digital literacy by offering training sessions or a telehealth patient navigator. 

Cancer patients in this study found telehealth visits to be easy to participate in (80%) and helpful (79%); they would recommend telehealth visits to other patients (87%). However, a relatively large proportion of them do not think telehealth is a good alternative to in-person visits (36%) and would not use telehealth if it were offered to them after the COVID-19 pandemic is over (37%). This mixed result aligns with previous work in this area. Some studies found that patients’ overall satisfaction with telehealth is high [9,10,12,13,14,15,16,17,18,19,20,21]. Reducing exposure to COVID-19 [15,22], including the time- and money-saving benefits of telehealth, promotes telehealth visits as a favorable option during the pandemic [10,16,17,20,22,23,24,43,44]. Despite its benefits, some patients felt that telehealth is not a good alternative to in-person visits because it does not optimize patients’ experience (e.g., no physical examinations) (43,44), and may compromise clinician-patient relationship, cybersecurity, and health care accessibility [12,25]. Moreover, the lack of emotional support, the varying levels of involvement of family and friends in care, the lack of resources and referrals, and being on active treatment have been shown to negatively affect patients’ satisfaction with telehealth [10,45]. 

Our results demonstrated that patients’ ratings of their experiences with telehealth reduced slightly during the COVID-19 pandemic. Before the pandemic, telehealth was perceived to be an add-on to their regular, in-person care. Patients were provided with the option of telehealth visits, resulting in a higher satisfaction with telehealth visits. The timing of our Wave 3 survey coincided with the third wave of the pandemic, when telehealth visits were more readily being offered to patients. The patients’ worse experiences with telehealth may be a result of not being provided the choice of in-person versus remote visits. 

The findings from our study further add to the evidence that telehealth may disproportionately benefit more advantaged populations. We found that patients with lower education levels and those who perceived telehealth to be difficult or very difficult had larger changes in their ratings of their telehealth experience. These findings are critical for future policy making and for developing health care infrastructure that ensures access to high-quality, person-centered care. Other sociodemographic factors such as race, age, culture, and immigration status have been shown to influence patients’ ability to have successful telehealth appointments, which in turn can worsen patients’ access to health services [9,25,27,46]. Previous studies have shown that during the pandemic, patients who were older, had shorter treatment duration, identified as a visible minority, were foreign born, were in linguistic isolation, or had recently immigrated had lower preferences for receiving telehealth for their cancer care [9,13,15,18,24,26].

In our study, females indicated lower preferences to use telehealth in the future; this is contrary to other reports that demonstrated that female cancer patients had greater satisfaction with telehealth during the COVID-19 pandemic [9,47]. These studies, however, did not focus on patients’ preferences for telehealth when provided with a choice between in-person and virtual visits in the future. This finding highlights that the possible sex and gender differences in the future uptake of telehealth visits need to be explored further using qualitative methodology to gain a richer understanding in intersectionality between the concepts of sex and gender. 

Our findings support the reduction of the rural-urban disparities in cancer care through the use of telehealth by improving health care access in rural communities [48,49]. Cancer-related symptoms (e.g., pain and discomfort) impose mental and physical hardship to patients, which may make traveling to access cancer care challenging. Patients from rural areas of BC have indicated high satisfaction with telehealth. They have a preference for video over phone visits and for telehealth as a supplement to rather than a replacement for in-person visits [50]. 

Our results showed that the mental health of cancer patients significantly increased during the pandemic. In line with our results, another longitudinal study found that HRQL of cancer patients was not affected as a result of the pandemic [51]. Given that COVID-19 restrictions applied to everyone with and without cancer, cancer patients did not feel they were missing out on anything by staying at home [52]. Moreover, they did not have to face difficult social interactions that required disclosing and explaining their diagnosis, which gave them a sense of relief and control [52]. Despite the anxiety, depression, and isolation following the COVID-19 pandemic, data suggest that some cancer patients appreciated the opportunity to stay at home and spend time with their families during the pandemic [52,53]. 

## 5. Limitations

This study contains a large number of patient responses regarding their telehealth experiences before and during the pandemic. However, patients providing survey responses may be subjected to selection bias, which may influence the overall generalizability of the results. Our data may not include populations that face a disproportionate burden of health and social inequities (e.g., unstable housing, food insecurity, mental health, and substance use challenges); therefore, their experiences of cancer care during the pandemic may not be captured as they may not have attended a telehealth visit or may not have participated in our survey. Our survey was only available in English or the three most commonly spoken non-English languages in BC. As such, it is possible that important and divergent perspectives of patients who could not complete the survey may have been missed. Furthermore, we did not verify the patients’ self-reported responses regarding whether they attended a telehealth visit during the pandemic; as a result, we have plans to link survey responses with BC Cancer health care utilization data. Wave 2 survey responses were collected between January and May 2020 for those patients receiving active treatment between July and October 2019; as such, patient responses to the Wave 2 survey may have been confounded by their experiences during the start of the pandemic. Our future work will include accessing survey responses from Wave 1 to have an unbiased before-pandemic sample. Further, conducting a longitudinal analysis will provide us with a better understanding of the evolution of patient experiences with telehealth. The study patients were also given the opportunity to provide written feedback regarding their experiences with telehealth in the survey, which we will analyze to gain a richer picture of cancer patients’ experiences. 

## 6. Conclusions

The increased use of telehealth during the pandemic allowed cancer patients access to their health care team while reducing their risk of exposure to COVID-19. While telehealth is associated with benefits, we found that patients’ experiences with telehealth were not equitable. The need to optimize cancer care and enhance equitable access to high-quality telehealth care is important as we navigate into post-pandemic cancer care. 

## Figures and Tables

**Table 1:** The characteristics of the study patients.

Demographics *	**N (%)**
All	1958 (100%)
**Age (years)**	
Mean (SD)	69.40 (10.98)
Min-Max	27–98
**Mental Component Summary**	
Mean (SD)	53.06 (10.81)
Min-Max	11.40–75.84
**Physical Component Summary**	
Mean (SD)	41.24 (11.85)
Min-Max	6.35–66.60
**Sex Assigned at Birth**	
Female	1074 (55%)
Male	884 (45%)
**Education**	
8th grade or less	65 (3%)
Some high school, but did not graduate	144 (4%)
High school or high school equivalency certificate	426 (22%)
College, CEGEP or other non-university certificate or diploma	553 (28%)
Post-graduate degree or professional designation	400 (20%)
Undergraduate degree or some university	358 (18%)
Missing	12 (1%)
**Race**	
White	1533 (78%)
East/Southeast Asian	237 (12%)
Mixed	38 (2%)
South Asian	24 (1%)
Middle Eastern	16 (1%)
Latino	15 (1%)
Indigenous (First Nations, Métis, Inuk/Inuit)	14 (1%)
Black	8 (0.4)
Other	31 (2%)
Prefer not to answer	36 (2%)
Missing	6 (0.3%)
**Health Authority**	
Fraser Health	589 (30%)
Vancouver Island Health	530 (27%)
Vancouver Coastal Health	420 (21%)
Interior Health	355 (18%)
Northern Health	64 (3%)
Region of Residence **	
Urban	1806 (92%)
Rural	152 (8%)
**Previous Experience with Telehealth**	
Yes	630 (32%)
No	1278 (65%)
Missing	50 (3%)
**Tumor Type**	
Breast	612 (31%)
Prostate	365 (19%)
Leukemia	117 (6%)
Multiple Myeloma	85 (4%)
Lung	75 (4%)
Other blood disorder	62 (3%)
Non-Hodgkin Lymphoma	52 (3%)
Bladder	50 (3%)
Cervix/uterine/ovarian/vulvar	40 (2%)
Colorectal	38 (2%)
Melanoma	19 (1%)
Kidney	17 (0.9%)
Liver	14 (0.7%)
Thyroid	14 (0.7%)
Non-invasive tumour	13 (0.7%)
Brain or central nervous system	10 (0.5%)
Pancreas	9 (0.5%)
Sarcoma	8 (0.4%)
Stomach	8 (0.4%)
Esophagus	7 (0.4%)
Oral	5 (0.3%)
Hodgkin Lymphoma	3 (0.2%)
Eye	2 (0.1%)
Testis	2 (0.1%)
Other	109 (6%)
Missing	222 (11%)

* If the value of a sociodemographic variable was missing from the Wave 3 data set, we took the value reported in Wave 2. ** If the second digit of patients’ postal code was a 0, then it was coded as rural; otherwise it was coded as urban.

**Table 2:** The changes in the frequencies of phone and video visits before and during the COVID-19 pandemic.

	Frequency before COVID-19	Frequency during COVID-19		
		None	1 Visit or More	Total
Phone visits *	None	308 (18%)	665 (39%)	973
	1 visit or more	177 (10%)	555 (33%)	732
	**Total**	485	1220	1705
Video visits **	None	1469 (82%)	146 (8%)	1615
	1 visit or more	87 (5%)	85 (5%)	172
	**Total**	1556	231	1798

* Frequency missing = 253; ** Frequency missing = 171.

**Table 3:** The patients’ experiences with telehealth visits during the COVID-19 pandemic.

How Easy or Difficult Was It for You to Participate in This Virtual Health/Telehealth Visit? *	N (%)	Was the Care Advice You Received during Your Virtual Health/Telehealth Visit(s) Helpful? *	N (%)
Very difficult	17 (1%)	Very unhelpful	81 (7%)
Difficult	32 (3%)	Unhelpful	25 (2%)
Neither easy nor difficult	188 (16%)	Somewhat helpful	141 (12%)
Easy	390 (33%)	Helpful	509 (45%)
Very easy	537 (46%)	Very helpful	382 (34%)
Missing	222	Missing	244

* Only patients who had telehealth visits were asked to answer these questions (*n* = 1382).

**Table 4:** The patients’ preferences for using telehealth.

	Do You Think Virtual Health/Telehealth Visits Could Be a Good Alternative to In-Person Visits for You in the Future?	Would You Use Virtual Health/Telehealth If Offered to You Your Care Providers When the COVID-19 Pandemic Is Over?	Would You Recommend Virtual Health/Telehealth to Other Patients? *
	N (%)	N (%)	N (%)
Definitely no	252 (14%)	225 (12%)	44 (4%)
Probably no	405 (22%)	453 (25%)	101 (9%)
Probably yes	761 (42%)	742 (41%)	545 (46%)
Definitely yes	391 (22%)	399 (22%)	487 (41%)
Missing	149	139	205

* Only patients who had telehealth visits were asked to answer this question (*n* = 1382).

**Table 5:** Patient characteristics affecting changes in telehealth experience ratings.

	ß	SE	Statistic	DF
Intercept	−2.759	1.611	−1.713	184.270
Age	−0.011	0.015	−0.725	165.699
Female	−0.082	0.288	−0.285	229.413
Urban	−0.168	0.387	−0.435	210.625
White	−0.083	0.443	−0.188	233.269
College or more **	−0.616	0.304	−2.030	213.659
Report of neither easy nor difficult participation *	2.010	1.021	1.969	76.195
Report of easy participation ***	3.717	0.927	4.011	90.480
Interior Health	0.535	0.520	1.030	130.482
Fraser Health	−0.244	0.611	−0.400	164.625
Vancouver Island	−0.012	0.356	−0.035	200.265
Northern Health	−0.565	0.539	−1.048	184.819
MCS in wave 2	−0.004	0.017	−0.247	171.569
PCS in wave 2	0.014	0.015	0.992	216.332
Change in MCS from wave 2 to 3	0.007	0.016	0.456	191.044
Change in PCS from wave 2 to 3	0.002	0.017	0.106	205.422

Abbreviations: DF, degree of freedom; MCS, Mental Component Score; PCS, Physical Component Score; SE, standard error. Referent categories for categorical variables: Sex, male; Region of residence, rural; Race, non-white; Education, high school or less; Perceived ease of participation, difficult participation; Health authority, Vancouver Coastal Health. * 0.05 < *p* < 0.1; ** 0.01< *p* < 0.05; *** *p* < 0.01.

**Table 6:** Patient characteristics affecting preference to use telehealth in the future.

	ß	SE	Odds Ratio (95% CI)
Intercept	−0.845	0.925	0.429 (0.069–2.644)
Age ***	−0.026	0.007	0.973 (0.961–0.987)
Previous experience with telehealth ***	0.375	0.135	1.456 (1.116–1.898)
Female **	−0.291	0.144	0.748 (0.564–0.992)
Urban *	−0.571	0.297	0.565 (0.315–1.012)
White *	0.317	0.174	1.373 (0.977–1.930)
College or more **	0.329	0.149	1.391 (1.036–1.867)
Report of neither easy nor difficult participation ***	2.430	0.601	11.359 (3.483–37.052)
Report of easy participation ***	3.393	0.588	29.751 (9.344–94.732)
Interior Health	−0.046	0.205	0.955 (0.639–1.429)
Fraser Health	−0.027	0.223	0.973 (0.628–1.508)
Vancouver Island	−0.055	0.207	0.947 (0.630–1.422)
Northern Health	−0.080	0.411	0.923 (0.412–2.069)
MCS in wave 3 **	0.0130	0.006	1.013 (1.001–1.025)
PCS in wave 3	−0.007	0.006	0.993 (0.982–1.005)

Abbreviations: CI, confidence interval; DF, degree of freedom; MCS, Mental Component Score; PCS, Physical Component Score; SE, standard error. Referent categories for the categorical variables: Previous experience, no; Sex, male; Region of residence, rural; Race, non-white; Education, high school or less; Perceived ease of participation, difficult; Health authority, Vancouver Coastal Health. * 0.05 < *p* < 0.1; ** 0.01 < *p* < 0.05; *** *p* < 0.01.

## Data Availability

The de-identified data supporting the findings of this article will be made available by the authors upon reasonable request.

## Supplementary Materials

The following supporting information can be downloaded at: https://www.mdpi.com/article/10.3390/curroncol29060335/s1, Figure S1: Proportion of phone (1a) and video (1b) visits with various health care professionals before and during the COVID-19 pandemic.
