# Supplementary material for: Cancer Patients’ Experiences with Telehealth before and during the COVID-19 Pandemic in British Columbia
Source: Curr Oncol. 2022 Jun 10;29(6):4199–211. doi: 10.3390/curroncol29060335 (PMC9222084; doi:10.3390/curroncol29060335)
Supplement: Supplementary file 1 [file curroncol-29-00335-s001.zip › curroncol-1728527-supplementary.pdf]

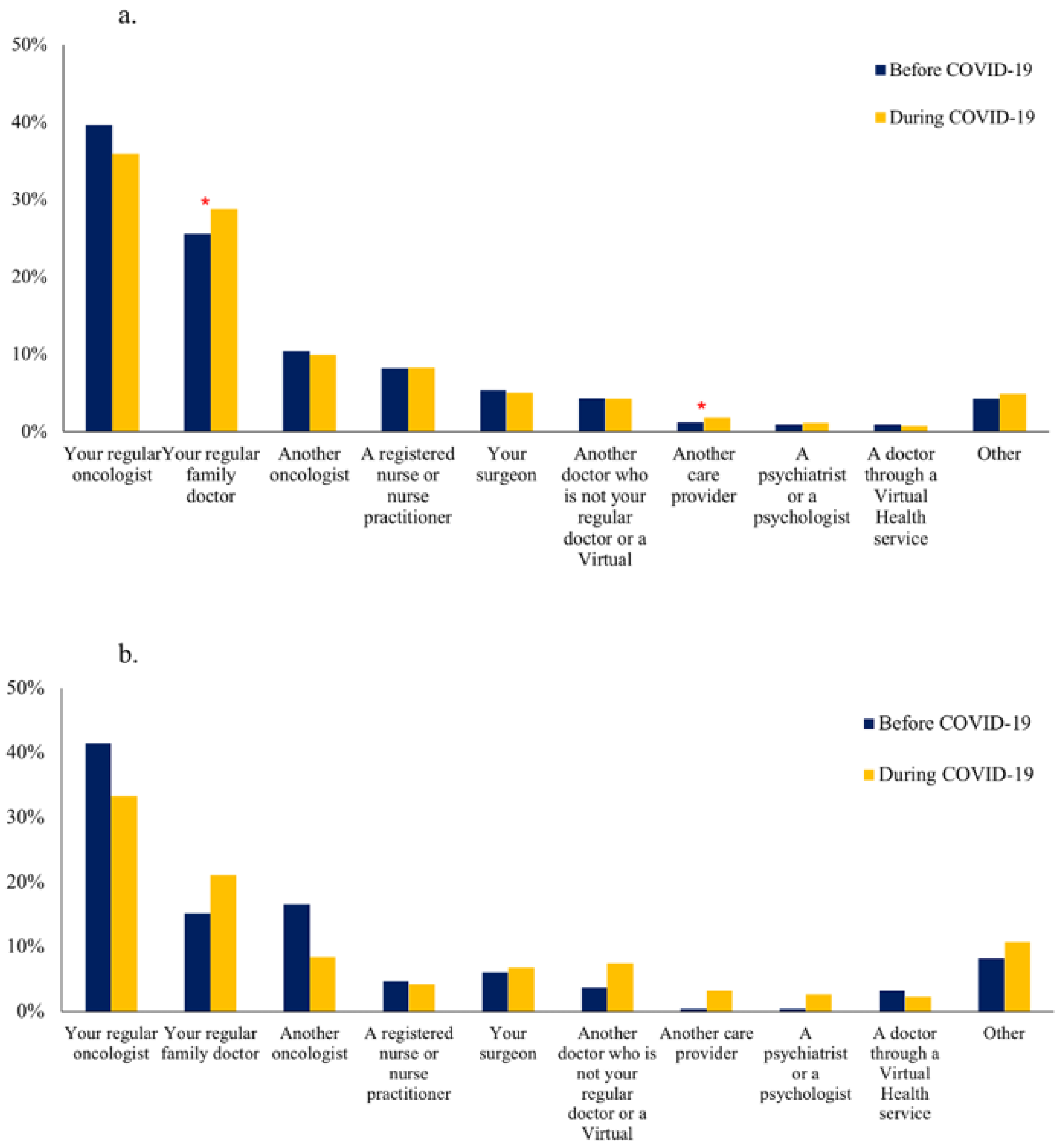

Figure S1. Proportion of phone (a) and video (b) visits with various healthcare professionals before and during the COVID-19 pandemic

*$p<0.05$
